# Peripheral canalicular branching is decreased in streptozotocin-induced diabetes and correlates with decreased whole-bone ultimate load and perilacunar elastic work

**DOI:** 10.1093/jbmrpl/ziad017

**Published:** 2024-01-04

**Authors:** Morgan W Bolger, Tara Tekkey, David H Kohn

**Affiliations:** Department of Biomedical Engineering, College of Engineering, University of Michigan, Ann Arbor, MI 48109, United States; Department of Chemistry, College of Literature, Science and the Arts, University of Michigan, Ann Arbor, MI 48109, United States; Department of Biomedical Engineering, College of Engineering, University of Michigan, Ann Arbor, MI 48109, United States; Department of Biologic and Materials Sciences, School of Dentistry, University of Michigan, Ann Arbor, MI 48109, United States

**Keywords:** osteocytes, bone histomorphometry, biomechanics, diabetes, lacunae, canaliculi

## Abstract

Osteocytes, the most abundant cell type in bone, play a crucial role in mechanosensation and signaling for bone formation and resorption. These cells reside within a complex lacuno-canalicular network (OLCN). Osteocyte signaling is reduced under diabetic conditions, and both type 1 and type 2 diabetes lead to reduced bone turnover, perturbed bone composition, and increased fracture risk. We hypothesized that this reduced bone turnover, and altered bone composition with diabetes is associated with reduced OLCN architecture and connectivity. This study aimed to elucidate: (1) the sequence of OLCN changes with diabetes related to bone turnover and (2) whether changes to the OLCN are associated with tissue composition and mechanical properties. Twelve- to fourteen-week-old male C57BL/6 mice were administered streptozotocin at 50 mg/kg for 5 consecutive days to induce hyperglycemia, sacrificed at baseline (BL), or after being diabetic for 3 (D3) and 7 (D7) wk with age-matched (C3, C7) controls (*n* = 10–12 per group). Mineralized femoral sections were infiltrated with rhodamine, imaged with confocal microscopy, then the OLCN morphology and topology were characterized and correlated against bone histomorphometry, as well as local and whole-bone mechanics and composition. D7 mice exhibited a lower number of peripheral branches relative to C7. The total number of canalicular intersections (nodes) was lower in D3 and D7 relative to BL (*P <* 0.05 for all), and a reduced bone formation rate (BFR) was observed at D7 vs C7. The number of nodes explained only 15% of BFR, but 45% of Ct.BV/TV, and 31% of ultimate load. The number of branches explained 30% and 22% of the elastic work at the perilacunar and intracortical region, respectively. Collectively, the reduction in OLCN architecture and association of OLCN measures with bone turnover, mechanics, and composition highlights the relevance of the osteocyte and the OLCN and a potential therapeutic target for treating diabetic skeletal fragility.

## Graphical Abstract

**Graphical Abstract:**
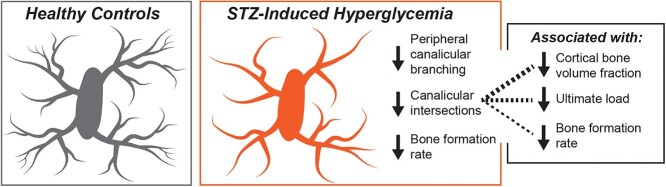

## Introduction

Osteocytes are the most abundant bone cells and are embedded throughout the mineralized matrix. Osteocyte cell bodies reside in lacunae, while the osteocyte dendrites exist within canaliculi. Together, the osteocytes, their dendritic extensions, and the surrounding structure form the osteocyte lacuno-canalicular network (OLCN), with connections to other osteocytes, bone-lining cells at the periosteal and endosteal surfaces, endothelial cells surrounding vasculature and, in osteonal bone, Haversian canal systems. Osteocytes play an active role in maintaining or remodeling their environment, and this maintenance or remodeling is affected by variety of triggers, including mechanical,^1^ hormonal (PTH, glucocorticoids,^2^ lactation,^3^ ovariectomy^4^), metabolic disease states,^5^^,^^6^ and aging.^7^ Osteocytes are also responsive to applied load and damage and rely on this OLCN to transmit signaling molecules such as sclerostin, DMP-1, and FGF-23^8^^,^^9^ to trigger bone formation or resorption by directly affecting osteoblast and osteoclast differentiation, recruitment, and proliferation.^10^

Patients with type 1 diabetes (T1D) have a 7-fold greater risk of hip fracture.^11^ Although patients with T1D have slightly lower BMD than age-matched healthy cohorts, this decrease in BMD is not enough to account for the increased fracture risk, suggesting other bone material properties are critical determinants of fracture.^11^^,^^12^ We have recently demonstrated that streptozotocin-induced hyperglycemia in mice led to perturbed perilacunar tissue mechanics and perilacunar collagen. Specifically, there was increased hardness, plastic index, and decreased creep distance in perilacunar bone adjacent to osteocytes compared to intracortical bone at a distance from osteocytes, and an increase in the perilacunar matrix maturity in the diabetic group vs control, overall indicating a disruption to the local tissue maintenance.^6^ Bone composition and material properties are partly dependent on bone turnover, which is decreased with T1D.^12^ Increases in osteocyte-derived sclerostin and RANKL with diabetes may decrease bone metabolism^13^ and coincide with perturbations to the OLCN. Specifically, mice induced to have type 2 diabetes (T2D) with a high-fat diet demonstrate increased osteocyte or lacunae volume and decreased node volume (volume of canalicular intersections).^5^ Akita mice, a T1D model, also exhibit decreased lacunar density.^14^

This study aimed to answer: (1) what is the temporal sequence of OLCN changes with diabetes, do these changes relate to decreased bone turnover, and (2) how do alterations in the OLCN affect local tissue composition and mechanical properties. The central hypothesis of this study was that diabetes in skeletally mature C57BL/6 mice will increase lacunae volume, decrease the number and interconnectivity of the canaliculi, and that these network changes will negatively correlate with decreased bone formation, increased plastic index, and increased matrix maturity.

## Materials and methods

### Animal study

This is a continuation of a recently published study where the same mice underwent a thorough characterization of local and whole-bone level mechanical and compositional characterization.^6^ Apart from markers of disease status (fasting blood glucose, HbA1c, weight), all data presented herein are new, as are the associations of OLCN and bone histomorphometry measures to prior measures of mechanical properties and tissue level composition. All animal procedures and protocols were conducted with approval from the University of Michigan Institutional Animal Care and Use Committee (IACUC). Briefly, single-housed male C57BL/6 mice 8–10 wk old were received for a baseline (BL) group, and 10- to 12-wk-old C57BL/6 male mice were received for the diabetic and control groups from Jackson Laboratory. The study was powered to detect changes in whole-bone mechanical properties after 7 wk. Only male mice were used as female mice do not exhibit the same susceptibility to streptozotocin-induced hyperglycemia.^15^^,^^16^ All animals at the 3-wk and 7-wk time points were age matched between the diabetic and control groups. Mice were allowed to acclimate for 2 wk and were then weight matched and administered five daily intraperitoneal doses of streptozotocin (STZ, Sigma Aldrich S0130) at 50 mg/kg in a sodium citrate buffer, while control mice received the sodium citrate buffer alone. Diabetes was defined as blood glucose >250 mg/dL and confirmed after a 4-h fasting period via blood measurements from tail veins (OneTouch Ultra2). If necessary, boosters of STZ at 50 mg/kg were administered until mice became diabetic. This experimental design resulted in 10–12 mice per experimental group, where the left femora (*n* = 10–12 per group) were imaged by microCT, then subjected to four-point bend. Half of the right femora (*n* = 5–6 per group) were infiltrated with rhodamine and imaged to characterize the OLCN, as later described, and the other half of the right femora group was imaged for histomorphometry, tested by nanoindentation, and then subjected to Raman spectroscopy. Fasting blood glucose and body weight were measured weekly. Investigators were blinded to end point measures but not for animal handling to ensure diabetic status was maintained. Mice were administered intraperitoneal alternating fluorochrome injections of calcein (15 mg/kg, Sigma C-0875) and alizarin complexone (25 mg/kg, Sigma A3882) 19, 13, 7, and 1 d prior to sacrifice. Mice were sacrificed after either 3 wk (D3) or 7 wk (D7) of diabetes, with age-matched controls (C3 or C7, respectively). At sacrifice, whole blood was collected for HbA1c measurements (A1CNow+, PTS Diagnostics), and serum was spun down and collected for bone turnover marker assays. All blood and serum were stored at −80 °C until use. Femora and tibiae were immediately harvested and stored at −80 °C in calcium-buffered soaked gauze.^17^

### Bone processing, staining, and imaging

Tissue allocation from this study and prior publication^6^ is outlined in Supplementary Figure 1. Fresh-frozen right femora (*n* = 10–12, per group) were embedded in polymethyl methacrylate (PMMA, Koldmount, SPI Supplies) and cured at room temperature overnight. This embedding procedure involved coating only the periosteal surface of the bone with a PMMA paste. Samples were subsequently sectioned, transversely, and no PMMA infiltrated the transverse face or OLCN. The OLCN was later cleared and subsequently stained with rhodamine. Embedded bones were then transversely cut at the midshaft with a low-speed circular diamond saw (South Bay Technology, Model 650). The proximal portion of the block was polished with a series of coarse to fine grit sandpaper (2400, 3000, and 4000), then polished with a 0.25-μm diamond suspension on a cloth, and subjected to a 5-min sonic bath in calcium-buffered PBS.^17^ This portion of the block was wrapped in calcium-buffered PBS and stored at −20°C prior to confocal imaging of the fluorochrome labels and later nanoindentation and Raman spectroscopy. From the distal half of the tissue block, adjacent to the middiaphysis, a 1-mm-thick section was cut using the low-speed circular diamond saw. The 1-mm sections (*n* = 6 per group) were fixed and cleared under vacuum with 70% ethanol overnight, then with 80%, 95%, and 100% ethanol, xylene, Clear-Rite 3 (Richard Allan Scientific), xylene, 100%, 95%, 80%, and 70% ethanol, each for 30 min. Rhodamine staining and imaging were adapted from published approaches.^18^^,^^19^ Sections were then incubated in 0.02% w/v rhodamine 6G (Sigma Aldrich, 83 697) in calcium-buffered PBS at room temperature, protected from light, with gentle agitation for 48 h. Sections were next rinsed three times with calcium-buffered PBS, mounted on glass slides, and polished with a series of coarse to fine grit sandpaper (2400, 3000, and 4000), then with a 0.25-μm diamond suspension on a cloth to ∼100 μm thickness. Slides were subjected to a 2,2′-thiodiethanol (TDE, Sigma Aldrich, 166 782) gradient of 10% TDE in PBS, followed by 25% and 50%, each for 30 min, then the slides were immersed overnight in 95% TDE. Samples were mounted with 1.5 glass coverslips in 97% TDE in PBS, which closely matches the refractive index of the immersion oil.^20^

Z-stack images for the OCLN morphology and topology analyses were acquired on a Nikon C2Plus confocal with a 60× oil objective, type F immersion oil, a 1.4-numerical aperture, 561/600 nm excitation/emission, and 60 pinhole radius. Z-stack images were acquired at 0.2 μm step size over a 30-μm image depth, 2048 × 2048 resolution. The imaged region of interest was the anterior quadrant of the femur, avoiding larger vessels or aberrations in the cortical bone. The first 2–5 visible μm were not imaged due to excessive rhodamine signal.

Z-stack images of lacunae were collected using the same sections on a Leica DMi8 Thunder imager microscope (Leica Systems), with a 63× oil objective, type F immersion oil, 1.4 numerical aperture, and acquired on a Leica DFC9000 camera at 2048 × 2048, in 540 MHz quality mode. Z-stacks had a 0.4-μm step size over the entire thickness of the section, at least 80 μm per section. Three z-stacks per section were acquired along the anterior quadrant. Each image was then subjected to large volume computational clearing, accounting for the refractive index of the 97% TDE in PBS of about ~1.51.

### OLCN morphology, topology, and lacunae morphology analysis

The acquired images of the OLCN were characterized in three ways: (1) examining the morphology of the OLCN—quantifying the number of canaliculi/lacunae and canalicular tortuosity; (2) quantifying lacunae morphology—lacunae volume, shape, and size; and (3) examining topology of the OLCN—skeletonizing the network to quantify connectivity.

**Figure 1:**
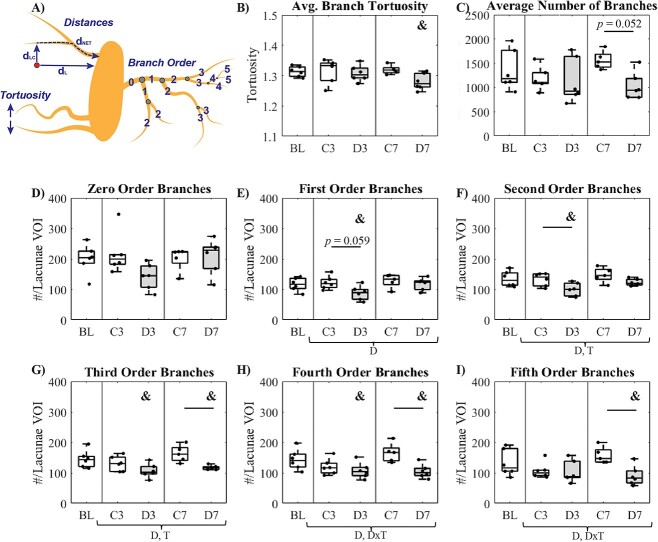
Osteocyte lacuno-canalicular network morphology measurements. (A) Distances indicated include the average distance from any point in the canalicular network to the lacunae through the OLCN (d_net_), the average shortest distance to lacunae through the bone matrix (d_L_), the average shortest distance from any point in the matrix to the network (d_LC_). Tortuosity is a measure of how convoluted of a path a canaliculi branch takes, with higher values indicating a more tortuous path. Branch order is assigned to individual branches of the canalicular network, where branches emanating off the lacunae are zero-order, and increase by one with each subsequent intersection. (B) Median number of branches around individual lacunae, (C) median branch tortuosity, (D–I) number of zero-order through fifth-order branches. For A, B, D–I, data are presented as boxplot with individual data points for each group for baseline (BL), 3-wk control (C3), 3-wk diabetic (D3), 7-wk control (C7), and 7-wk diabetic (D7). Two-way ANOVA carried out for C3, D3, C7, and D7 and significance (*P* < .05) between groups after Tukey’s post hoc is indicated by horizontal bars. Significant factor effects (*P* < .05) for diabetic status (D), time (T), and diabetic status–time interaction (D × T) are indicated below the *x*-axis. One-way ANOVA with least-squares difference post hoc was carried out to compare for significant differences relative to BL indicated by **&** (*P* < .05).

OLCN morphology analysis was conducted using AIVIA 10.0 software (Leica Microsytems). After importing the z-stack .nd2 output files from the confocal, 200 × 200 × 120 pixel (x,y,z) volumes of interest were centered on individual lacunae that were entirely captured in the 30 μm section (∼5–6 VOI per z-stack). The 3D Neuron Analysis package within AIVIA was used, isolating these volumes of interest, and allowing for automatic soma (lacunae) and dendrite (canaliculi) detection. Accuracy of lacunae and canaliculi detection against the rhodamine-detected channel was conducted visually, and the Neuron Analysis detection parameters were adjusted accordingly to ensure that the detection program captured all canaliculi present in the rhodamine channel and did not overestimate the number of canaliculi. OLCN morphology measurements automatically calculated on the individual detected lacunae and canaliculi included: branch count, individual branch order, maximum child branch order, mean branch tortuosity, and branch length (Figure 1A).

On the same z-stack images, a pixel classifier recipe was taught and iteratively improved to assign a probability that each pixel belonged to the OLCN network. This channel output was then processed in ImageJ.^21^ Images were reduced to 8-bit, converted from 2048 × 2048 to 1024 × 1024, rotated so that the anterior endosteal and periosteal surfaces ran parallel to the x-axis, brightness and contrast adjusted, and a 500 × 500 × 100 pixel VOI was then cropped in regions with a consistent number of lacunae, avoiding vasculature or other aberrations. This output was then analyzed on Matlab 2022a (Mathworks) using code previously written^19^ and adapted^22^ to convert images into skeletonized maps for topology outputs. Images were analyzed in parallel pools on an HP Z800 workstation, Intel Xeon 3.20 GHz, 192 GB RAM, and 64-bit OS. The measured outputs included the number of nodes (canalicular intersections) and edges (connections between nodes), as well as the average distance from any point in the canalicular network to the lacunae through the OLCN (d_net_), the average shortest distance to lacunae through the bone matrix (d_L_), and the average shortest distance from any point in the matrix to the network (d_LC_) (Figure 1A). Additionally, the number of cluster nodes (C-nodes) which have a clustering coefficient of > 0.5, and the number of tree-type nodes with a clustering coefficient of 0 and edge count of 3 (T-nodes) were calculated. A clustering coefficient is a measure of node connectivity, a value of 1 indicates that all neighboring nodes are connected to each other, while 0 indicates that no neighboring nodes are connected. Edge count is the number of offshoots from a particular node to another node.

Z-stack images acquired on the Leica Thunder microscope were used to characterize the lacunae morphology using open-source code^23^ on Matlab 2022a (Mathworks). Two to three z-stacks taken on the anterior mid-cortex of the bone were analyzed per bone (*n* = 6 per group) and at least 60 lacunae were captured per z-stack. Calculated outputs include direct measurements of lacunae sphericity, radius, and volume, as well as the same measurements for a fitted ellipsoid.

### Histomorphometry and serum turnover markers

Polished proximal blocks of the femora (*n* = 10–12 per group) were imaged on a Nikon C2Plus confocal with a 20× objective, using the automated stitching software. Histomorphometry analysis followed the guidelines set by the American Society for Bone and Mineral Research^24^ and was completed on ImageJ using the RGB Profiler to measure interlabel distance. All histomorphometry analyses were conducted on the posterior periosteal surface. Measures calculated include percent mineralizing surface (MS/BS = (double-labeled surface + single-labeled surface/2)/bone surface), mineral apposition rate (MAR = interlabel distance/time), and bone formation rate (BFR = MAR × MS/BS). At the endpoint, thawed serum samples were collected and separated. Levels of the bone formation marker N-terminal propeptide of type I procollagen (P1NP) and the bone resorption marker C-terminal telopeptide of type I collagen (CTX) were then measured using commercially available kits (AC-33F1, AC-06F1, Immunodiagnostic Systems).

### Whole-bone mechanics, morphology and local Nanoindentation, Raman spectroscopy

The whole-bone mechanical properties, cross-sectional morphology, and local measures, conducted using nanoindentation and Raman spectroscopy, were previously reported, along with their detailed methodology.^6^ Briefly, femora were imaged using a μCT (μCT100 Scanco Medical), and 2D morphology and 3D density, as well as volume-based measurements, were calculated, including total volume (TV), bone volume (BV), bone volume fraction (BV/TV), BMD, and tissue mineral density (TMD), and cortical cross-sectional area (CA). Bones were then subjected to four-point bending on an eXpert 450 Universal Testing Machine (Admet) with a 0.01-mm/s loading rate, and the anterior surface in tension. The 0.2% offset method was used to calculate the yield point.

On the same proximal blocks that were imaged for histomorphometry, subsequent nanoindentation, followed by Raman spectroscopy, were conducted to characterize the local perilacunar and intracortical composition and mechanical properties. Measurements were taken at two locations in the anterior quadrant: (1) perilacunar: less than 5 μm from a lacunae wall and (2) intracortical: greater than 20 μm from any visible lacunae wall. The nanoindentation loading scheme included a 60-s hold to assess creep behavior, with creep distance being extracted from the creep holding period. From the entire loading/unloading curve, calculations were performed for plastic and elastic work, as well as the plastic index. Modulus and hardness values were determined from the unloading curve. Nanoindentation outcomes were not corrected for mineral content. Raman spectroscopic measurements were taken following the same location criteria as nanoindentation, but care was taken to avoid indented sites. Previously indented sites were clearly visible on the surface of the bone, and Raman measurements were taken on the opposite side of the lacunae (Supplementary Figure 1). Specific bands of interest were the phosphate band at ~959 cm^−1^, carbonate at ~1070 cm^−1^, proline at ~853 cm^−1^, and hydroxyproline at ~876 cm^−1^. Bands of interest from the Amide I region were ~1660 cm^−1^ and ~1690 cm^−1^. Calculated measurements include the proline/hydroxyproline ratio (876/853 cm^−1^), crystallinity (1/FWHM 960 cm^−1^), mineral-to-matrix (MMR, 960/[876 + 853 cm^−1^]), carbonate/phosphate (1070 /959 cm^−1^), and the Amide I 1660/1690 cm^−1^ ratio. This 1660/1690 cm^−1^ ratio has been demonstrated to reflect differences in the ratio of mature to immature cross-links^25^^,^^26^ and potential changes in collagen secondary structure.^27–29^

### Statistics

**Figure 2:**
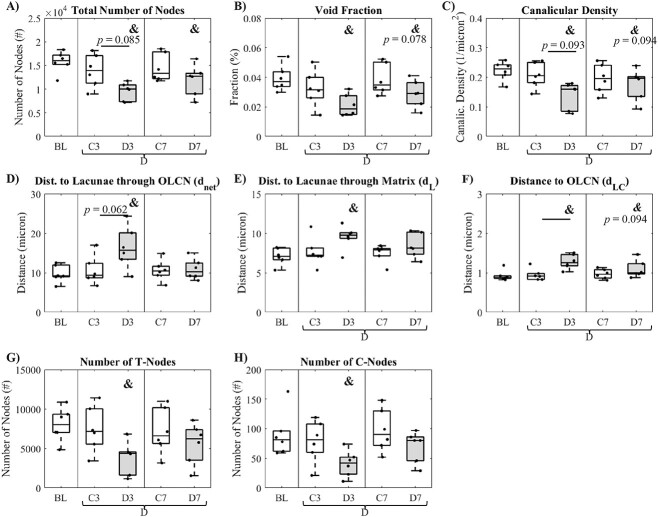
Osteocyte lacuno-canalicular topology. (A) Total number of nodes in the network volume of interest, (B) void fraction of the network, (C) canalicular density, (D) average distance from any point to a lacunae through the network (d_net_), (E) shortest distance to a lacunae through the bone matrix (d_L_), (F) average distance from any point to the canalicular network (d_LC_), (G) number of T-nodes, and (H) number of C-nodes. Data are presented as boxplot with individual data points for each group for baseline (BL), 3-wk control (C3), 3-wk diabetic (D3), 7-wk control (C7), and 7-wk diabetic (D7). Two-way ANOVA was carried out for C3, D3, C7, and D7 and significance (*P* < .05) between groups after Tukey’s post hoc is indicated by horizontal bars. Significant factor effects (*P* < .05) for diabetic status (D), time (T), and diabetic status–time interaction (D × T) are indicated below the *x*-axis. One-way ANOVA with least-squares difference post hoc was carried out to compare for significant differences relative to BL indicated by **&** (*P* < .05).

**Figure 3:**
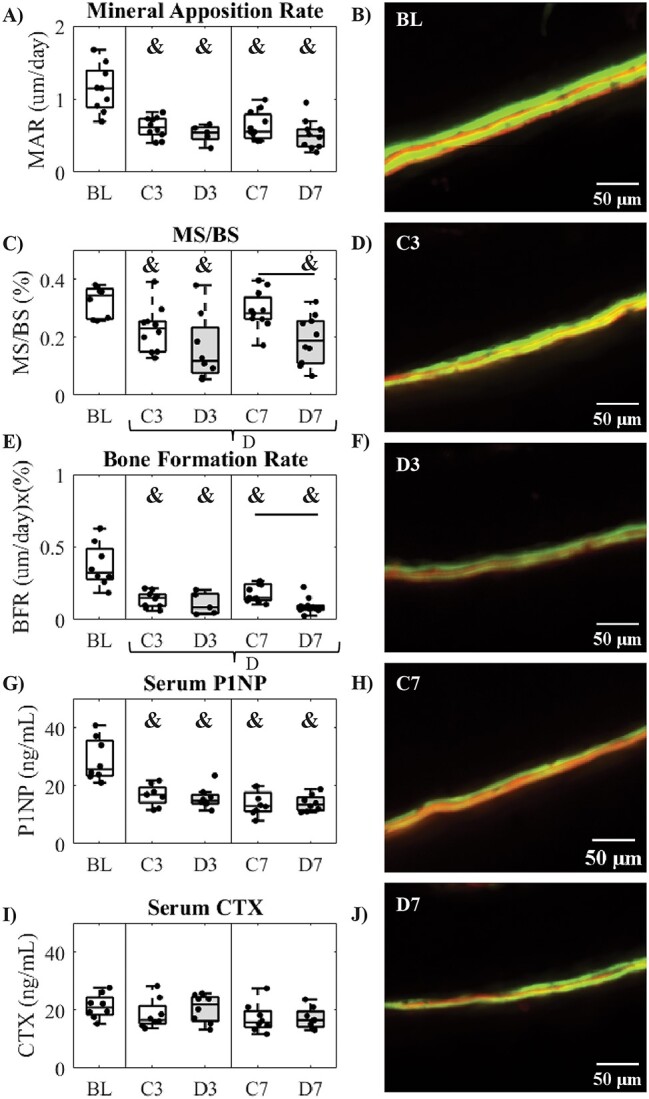
Histomorphometry and serum markers. (A) Mineral apposition rate, (B) mineralizing surface/bone surface (MS/BS), (C) BFR, (D) serum P1NP concentration, (E) serum CTX concentration, (F–J) representative images of labels in the periosteal posterior quadrant. Data are presented as mean with individual data points for each group for baseline (BL), 3-wk control (C3), 3-wk diabetic (D3), 7-wk control (C7), and 7-wk diabetic (D7). Two-way ANOVA was carried out for C3, D3, C7, and D7, and significance (*P* < .05) between groups after Tukey’s post hoc is indicated by horizontal bars. Significant factor effects (*P* < .05) for diabetic status (D), time (T), and diabetic status–time interaction (DxT) are indicated below the *x*-axis. One-way ANOVA with least-squares difference post hoc was carried out to compare for significant differences relative to BL indicated by **&** (*P* < .05).

**Figure 4:**
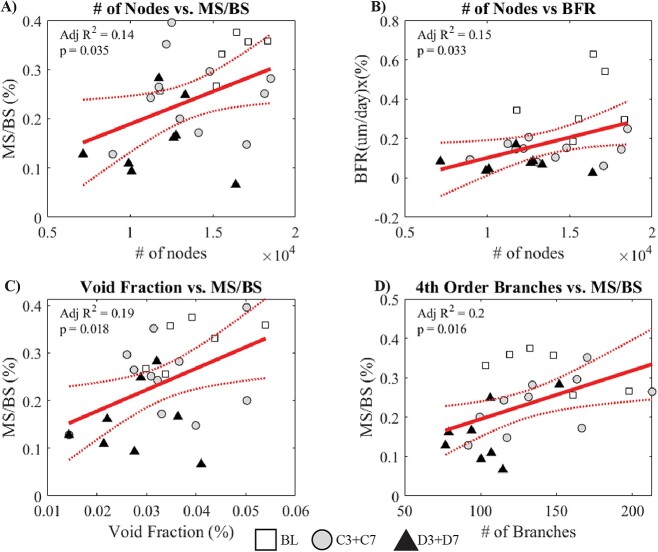
Key linear regressions testing how osteocyte lacuno-canalicular parameters relate to measures of bone formation. (A) Average number of nodes vs MS/BS, (B) average number of nodes vs BFR, (C) void fraction vs MS/BS, (D) average number of fourth-order branches vs MS/BS. Linear fits are presented alongside 95% confidence intervals, with individual data points, and adjusted *R*^2^ with *P-*values.

**Figure 5:**
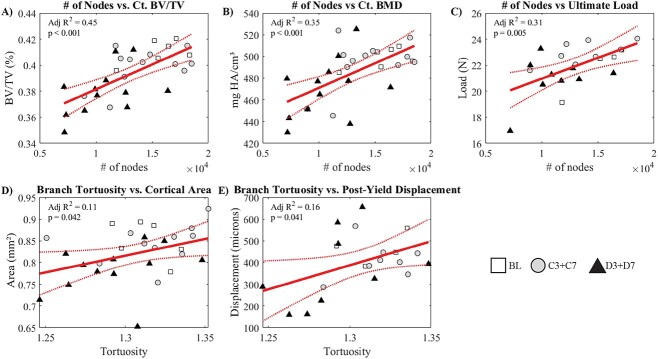
Key linear regressions testing how osteocyte lacuno-canalicular parameters relate to whole-bone measures of morphology, density, and mechanical properties. (A) Average number of nodes vs Ct. BV/TV, (B) average number of nodes vs Ct.BMD, (C) average number of nodes vs ultimate load, (D) average branch tortuosity vs cortical area, (E) average branch tortuosity vs postyield displacement. Linear fits are presented alongside 95% confidence intervals, with individual data points, and adjusted *R*^2^ with *P-*values.

**Figure 6:**
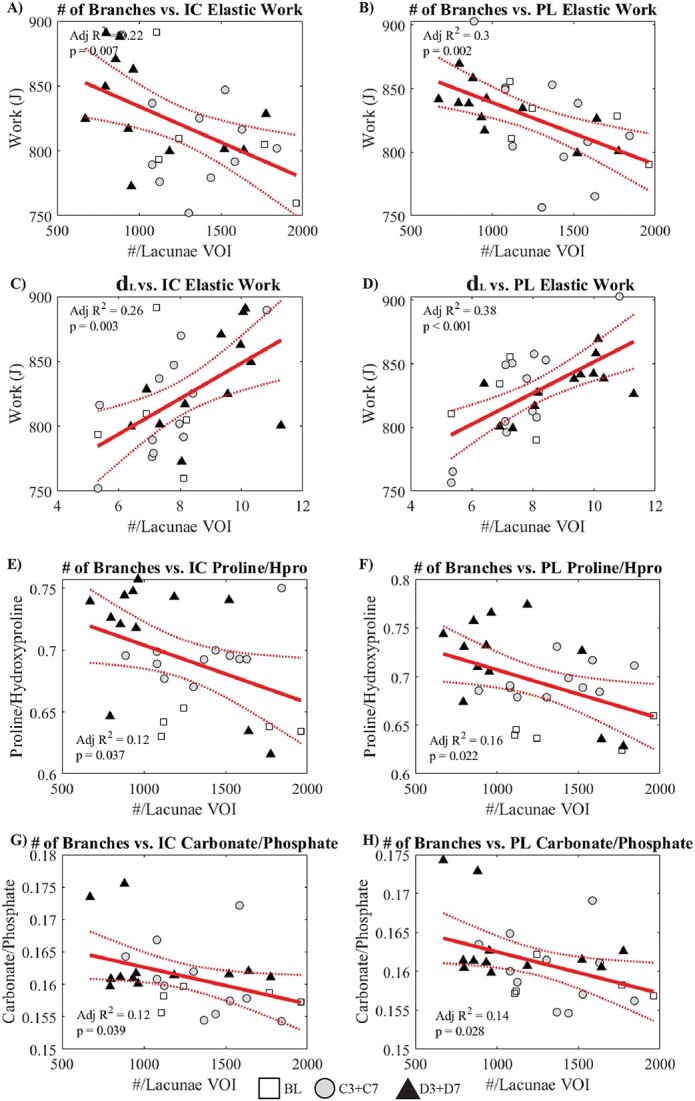
Key linear regressions testing how osteocyte lacuno-canalicular parameters relate to local Raman spectroscopic and nanoindentation measurements. (A) Average number of # of branches vs intracortical elastic work, (B) average number of # of branches vs perilacunar elastic work, (C) d_L_ vs intracortical elastic work, (D) d_L_ vs perilacunar elastic work, (E) average number of # of branches vs intracortical proline/hydroxyproline, (F) average number of # of branches vs perilacunar proline/hydroxyproline, (G) average number of # of branches vs intracortical carbonate/phosphate, (H) average number of # of branches vs perilacunar carbonate/phosphate. Linear fits are presented alongside 95% confidence intervals, with individual data points, and adjusted *R*^2^ with *P-*values.

All statistical analyses were carried out in Matlab 2021a (Mathworks). To test for differences between C3, D3, C7, and D7, two-way ANOVAs with Tukey’s post hoc tests were conducted, and factor effects for diabetes, time, and their interaction were noted. One-way ANOVAs with Fisher’s least significant difference post hoc tests were conducted to compare BL to C3, D3, C7, and D7. Significance for all tests was defined as *P* < 0.05, with trending cases of *P* < 0.10 noted in the figures with their corresponding *P-*value. Data in Figures 1–3 are presented as boxplots overlaid with individual data points. Supplementary Table 1 data are presented as mean (standard deviation) for each group. We hypothesized that OLCN measures would be predictive of bone turnover, local composition, and whole-bone and local mechanical properties. To test this hypothesis, Pearson correlation coefficients were calculated, and significant (*P* < 0.05) coefficients are presented in Table 1 and Supplementary Tables 2 and 3. Linear regressions were conducted on key traits where significant Pearson correlations were detected, and Figures 4–6 present these linear regressions with their adjusted *R*^2^ values and *P-*values noted, as well as the 95% confidence intervals.

**Table 1:** Pearson correlation coefficients between osteocyte lacuno-canalicular parameters and bone markers. Only significant correlations (*P* < .05) area included in the table.

	**Average blood glucose**	**HbA1c**	**MS/BS**	**BFR**	**P1NP**	**CTX**
**Average blood glucose**		0.92	−0.51	−0.46	−0.39	
**HbA1c**	0.92		−0.43	−0.45	−0.37	
**# of nodes**	−0.50	−0.42	0.42	0.43		
**Void fraction**	−0.40		0.47			
**Canalicular density**	−0.44	−0.39				
**d**_**net**_						
**d**_**L**_			−0.44			
**d**_**LC**_	0.54	0.45	−0.43			
**# of T-nodes**	−0.46	−0.39				
**# of C-nodes**	−0.42		0.48			
**Average # of branches**						
**Average branch tortuosity**						
**# of zero-order branches**						
**# of first-order branches**	−0.41					
**# of second-order branches**	−0.52		0.43			
**# of third-order branches**	−0.52		0.51			
**# of fourth-order branches**	−0.46		0.49			
**# of fifth-order branches**	−0.38					

## Results

### STZ-induced diabetes increased blood glucose, hemoglobin, and prevented weight gain

Animals from this study were included in a previously published study.^6^ Apart from quantification of the disease state (weight, fasting blood glucose, HbA1c), all comparisons of OLCN and bone histomorphometry between groups are new. Although group differences in composition and mechanics were previously published, the associations between OLCN or histomorphometry vs composition and mechanical outcomes are novel.

Their diabetic status was previously quantified,^6^ but the disease status of only the animals used in this study (Supplementary Figure 1, *n* = 10–12 per group) is described herein. Streptozotocin administration increased the fasting blood glucose of D3 (328 ± 42 mg dL^–1^) and D7 (358 ± 80 mg dL^–1^) relative to age-matched controls C3 (152 ± 15 mg dL^–1^), C7 (156 ± 9 mg dL^–1^), and BL (158 ± 18 mg dL^–1^, *P* < 0.001 for all). No difference was detected between either control C3 or C7 and BL (*P* = 0.844, *P* = 0.900). Significant factor effects were observed for diabetes (*P* < 0.001). Similarly, for HbA1c, both D3 (6.1 ± 0.6%) and D7 (7.0 ± 1.4%) exhibited higher HbA1c levels compared to their age-matched controls C3 (4.5 ± 0.2%), C7 (4.7 ± 0.2%), and BL (4.5 ± 0.2%, *P* < 0.001 for all). The HbA1c levels of D7 were also higher than D3 (*P =* 0.014), with significant factor effects for diabetes (*P* < 0.001) and time (*P =* 0.017).

Mouse body weight was significantly lower in D3 (27.7 ± 1.6 g) and D7 (27.4 ± 2.1 g) mice relative to their controls C3 (30.9 ± 2.9 g, *P =* 0.005) and C7 (30.1 ± 1.8 g, *P =* 0.002), with no difference in D3 or D7 vs BL (26.8 ± 2.4 g, *P* = 0.328, *P =* 0.512). However, both C3 and C7 weighed more than BL (*P <* 0.001, *P =* 0.001). Factor effects were significant for diabetes (*P* < 0.001).

### Diminished OLCN at the periphery of the network with diabetes

Tortuosity is a measurement of how convoluted a path is between two points (Figure 1A). Although no significant difference between control and diabetic groups existed for branch tortuosity (Figure 1B), D7 mice did exhibit a significant decrease in branch tortuosity relative to BL (*P =* 0.041). The average total number of branches around each lacunae (Figure 1C) exhibited a borderline decrease in D7 mice compared to C7 mice (*P* = 0.052). Branch order increases with each bifurcation away from the lacunae (Figure 1A). No significant difference (Figure 1D) between groups was detected for zero order branches (or # of branches originating from the lacunae), butD3 mice exhibited significantly fewer first-order branches (Figure 1E) than BL mice (*P =* .031) with a diabetic factor effect (*P =* .024). D3 mice also possessed significantly fewer second-order branches compared to BL and C3 mice (Figure 1F, *P=* .010, *P =* .044), with significant diabetic (*P =* .003) and time (*P =* .037) factor effects. D3 and D7 mice exhibited a significantly lower number of third-order branches (Figure 1G) relative to BL mice (*P =* .008, *P =* .048), and D7 mice had fewer branches relative to C7 mice (*P =* .012) with significant diabetic (*P =* .001) and time (*P =* .027) factor effects. Similarly, D3 and D7 mice showed a significantly lower number of fourth-order branches (Figure 1H) relative to BL (*P =* .034, *P =* .024), and fewer in D7 mice relative to C7 mice (*P =* .006) with significant diabetic (*P =* .004) and interaction (*P =* .041) factor effects. The number of fifth-order branches was significantly lower in D7 mice relative to BL (*P =* .043) and C7 (*P =* .010), with significant diabetic (*P =* .013) and interaction (*P =* .024) factor effects. No significant difference was detected between the number of bifurcations or average branch path length between any of the groups (data not shown).

Examining the OLCN as a topological map, the total number of nodes (Figure 2A) in D3 and D7 mice was significantly lower compared to BL mice (*P <* .001, *P =* .027) and a significant factor effect for diabetes existed (*P =* .010). The network void fraction (the fraction volume occupied by the OLCN) in D3 mice was significantly lower than BL (*P =* .003) and a significant factor effect for diabetes was detected (Figure 2B, *P =* .018). The canalicular density (Figure 2C) was significantly lower in D3 mice relative to BL mice (*P =* .004) with a significant factor effect for diabetes (*P =* .036). D3 mice exhibited an increase relative to BL mice in d_net_—the average distance from any point to the lacunae through OLCN—(Figure 2D, *P =* .003). Additionally, the average shortest distance to lacunae through the bone matrix (d_L_) was significantly greater for D3 mice relative to BL mice (Figure 2E, *P =* .006) with a significant diabetic factor effect (*P =* .032). D3 mice also exhibited a significant increase in the average distance from any point in the matrix to the network (d_LC_, Figure 2F) relative to BL (*P* < .001) and C3 mice (*P* = .015) with a significant diabetic factor effect (*P =* .004). No significant differences or factor effects were detected for mean node degree or the average number of offshoots from a single node (data not shown). D3 mice exhibited a significantly lower number of T-nodes relative to BL mice (Figure 2G, *P**=* .008) with a significant diabetic factor effect (*P* = .026). The number of C-nodes was significantly lower in D3 mice relative to BL (Figure 2H, *P* = .013) and C7 mice (*P* = .022), with a significant diabetic factor effect (*P* = .017). However, no significant difference between groups or factor effects was detected for the percentage of T- or C-nodes when normalized to total node count (data not shown). Collectively, the network analyses demonstrated that the OLCN with diabetes was sparser in comparison to age-matched controls.

No significant differences were detected in any lacunae morphology parameter (Supplementary Table 1), including lacunae density, lacunae void fraction, surface area, volume, surface area, ellipsoidal surface area, volume, sphericity, span theta, or radius. C3, D3, and D7 mice did exhibit an increase in distance to the nearest closest center of mass relative to BL mice (*P =* .012, *P =* .035, *P =* .018). No factor effects were detected for any lacunae morphology trait.

### Bone formation is reduced with STZ-induced diabetes

All groups of mice (C3, D3, C7, and D7) exhibited significantly lower MAR relative to baseline (Figure 3A, *P* < .001 for all). Percent mineralizing surface (MS/BS, Figure 3C) was significantly decreased in C3, C7, and D7 mice relative to BL (*P =* .017, *P <* .001, *P =* .001) and in D7 mice relative to C7 mice (*P =* .047) with a significant diabetic factor effect (*P =* .004). BFR (Figure 3E) was significantly lower in all groups relative to BL (*P* < .001 for all). D7 mice also exhibited a lower BFR relative to C7 mice (*P =* .011) with a diabetic factor effect (*P =* .008). All groups demonstrated lower serum levels of P1NP (Figure 3G) relative to BL (*P* < .001 for all). No significant differences or factor effects were detected between groups for serum CTX (Figure 3I), and the P1NP/CTX ratio followed the P1NP trends, with all groups lower than baseline (data not shown).

### OLCN parameters strongly correlate with measures of diabetic status

To better understand the relationship between the OLCN, disease status, and bone formation, correlation and linear regression analyses were carried out. Significant (*P* < .05) Pearson correlation coefficients for OLCN traits are included in Table 1. To contextualize OLCN differences with the diabetic state, many traits negatively correlated with both average fasting blood glucose and HbA1c, including the number of nodes, canalicular density, and number of T-nodes. Higher blood glucose and HbA1c positively correlated with higher average distance from any point in the matrix to the OLCN (d_LC_). Traits that negatively correlated with fasting blood glucose, but not HbA1c, included network void fraction, the number of C-nodes, and number of first-order through fifth-order branches. MS/BS negatively correlated with the average distance from any point in the matrix to the lacunae wall (d_L_) and average distance from any point in the matrix to the OLCN (d_LC_), and positively with the number of nodes, void fraction, number of C-nodes, and number of second-order through fourth-order branches. The only OLCN trait that positively correlated with BFR was the number of nodes, and no correlation existed between any OLCN trait and serum markers of bone turnover, P1NP and CTX.

The number of nodes explained ∼14% of the variance in MS/BS and 15% in BFR (Figure 4A, *P* = .035; 4B, *P* = .033). The void fraction accounted for ∼19% of the variance in MS/BS (Figure 4C, *P* = .018), whereas the number of fourth-order branches explained 20% of the variance in MS/BS (Figure 4D, *P* = .016).

### The number of nodes has explanatory power over cortical morphology and ultimate load

Significant Pearson coefficients (*P* < .05) comparing the OLCN parameters against whole-bone cortical morphology and mechanical properties are listed in Supplementary Table 2. The average number of nodes, canalicular density, number of T- and C-nodes, and the number of second-order through fourth-order branches all showed positive correlations with both Ct.BV/TV and Ct.BMD. In contrast, d_L_ and d_LC_ both negatively correlated with Ct.BV/TV and Ct.BMD. No traits correlated with Ct.TMD- or total CA. The average number of nodes, canalicular density, number of T-nodes, branch tortuosity positively correlated with cortical area, and d_LC_ correlated negatively with cortical area. The average number of nodes, canalicular density, number of C- and T-nodes, and zero-order through second-order branches all positively correlated with ultimate load, while d_LC_ negatively correlated with ultimate load. Summarizing, OLCN parameters, such as the number of nodes, were predictive of whole-bone density, geometry, and ultimate load.

The average number of nodes explained ∼45% of the variance in Ct.BV/TV (Figure 5A, *P* < .001), 35% of the variance in Ct.BMD (Figure 5B, *P* < .001), and 31% of the variance in ultimate load (Figure 5C, *P* = .005). The average branch tortuosity explained ∼11% of the variance in cortical area (Figure 5D, *P* = 0.042) and 16% of postyield displacement (Figure 5E, *P* = .041). The regressions demonstrate that OLCN parameters, such as number of nodes, increased with increases in measures of whole-bone cortical morphology (Ct.BV/TV, Ct.BMD) and ultimate load, indicating that a more intact osteocyte network correlates with increased mechanical properties at the whole-bone level.

### The number of branches is predictive of local elastic work and composition, with stronger associations at the perilacunar site compared to intracortical

OLCN parameters were correlated against local Raman spectroscopic and nanoindentation measures taken at the intracortical and perilacunar regions (Supplementary Table 3). Overall, the OLCN parameters demonstrated greater explanatory power over composition (proline/hydroxyproline, carbonate/phosphate) and mechanical measures (elastic work) closer to the lacunae in the perilacunar region than the intracortical region.

Canalicular density, number of C-nodes, average number of branches, average branch tortuosity, and the number of fifth-order branches all negatively correlated with perilacunar elastic work. While d_net,_ and d_L_ positively correlated with perilacunar elastic work. At the intracortical region, only d_L_ positively correlated with elastic work and the average number of branches, and fifth-order branches negatively correlated with elastic work (Supplementary Table 3).

At both the intracortical and perilacunar regions, the proline/hydroxyproline ratio negatively correlated with the average number of branches, but only negatively correlated with branch tortuosity for the intracortical region. Similar relationships existed for carbonate/phosphate, which negatively correlated with the average number of branches and second through fourth order branches for both regions. Mineral crystallinity positively correlated with the number of fourth-order branches for both the IC and PL regions, and the PL region had additional significant positive correlations between mineral crystallinity and the number of third- and fifth-order branches. The Amide I 1660/1690 cm^−1^ ratio negatively correlated with average branch tortuosity for the perilacunar region only (Supplementary Table 3).

Synthesizing the linear regressions, the number of branches explained ∼22% of the variation in elastic work at the intracortical region (Figure 6A) and 30% of the variance at the perilacunar region (Figure 6B). The distance from any point in the matrix to the lacunae explained 26% of the variance in intracortical elastic work (Figure 6C) and 38% of the variance in perilacunar elastic work (Figure 6D). Compositional traits provided lower explanatory power. The number of branches in the lacunae volume of interest explained 12% of the intracortical proline/hydroxyproline (Figure 6E) and 16% of the perilacunar proline/hydroxyproline (Figure 6F). The number of branches also explained 12% (Figure 6G) and 14% (Figure 6H) of the carbonate/phosphate at the intracortical and perilacunar regions, respectively.

## Discussion

This study examined perturbations of the osteocyte lacunar–canalicular network in a hyperglycemic setting and established relations between changes in OLCN and local perilacunar composition and mechanical properties. The most profound changes in OLCN morphology were the decreased number of third-order through fifth-order branches following 7 wk of diabetes (Figure 1). A decrease in the number of first-order and second-order branches also occurred after 3 wk of disease. As these measurements were taken at already embedded osteocytes, the decrease in branch order suggests that under a diabetic state, the maintenance of peripheral canaliculi is diminished, and osteocyte dendritic extensions may be retracting. There was also a significantly lower number of nodes (Figure 2A) and trends toward decreased void fraction and canalicular density after 7 wk of diabetes (Figures 2B and 3C) vs baseline, with significant diabetic factor effects for the number of nodes, void fraction, canalicular density, shortest distance to lacunae through matrix, and distance to the OLCN—all of which indicate a sparser network. The reduction in higher order branches, reduction in nodes, and trending decrease of canalicular density with diabetes (Figures 1 and 2) is consistent with age-related reductions in dendrite number, which precede age-related decreases in osteocyte number^30^ and reduced nodes in an osteocyte-intrinsic TGF-β knockout in which perilacunar remodeling is arrested.^22^

The diminished OLCN morphology and topology were detectable at 7 wk of diabetes, with some outcomes, including decreased canalicular density, number of nodes, and increased distance to the OLCN being more prominent at 3 wk than at 7 wk. Although the changes in D3 but not in D7 mice highlight the biological variance, the compromises to the OLCN following 3 wk of diabetes preceded the decrease in BFR in D7 (Figure 3), supporting the hypothesis that the changes to the OLCN and perilacunar remodeling with diabetes precede reductions in bone formation. Additionally, several differences were measured in D3 or D7 vs BL but not compared to their age-matched controls. There was a lower number of first-, third-, and fourth-order branches, lower total node count, void fraction, canalicular density, number of T-nodes and number of C-nodes in D3 relative to BL but not to C3. There was also a lower number of total nodes and lower branch tortuosity in D7 vs BL but not C7. This suggests that there may be slight age-related diminishments in OLCN architecture between 10 and 15–19 wk, with possible superposition of aging and disease effects.

A thorough recent review has summarized the effect of different physiological conditions on osteocyte viability, OLCN morphology, local tissue composition, and mechanical properties.^31^ However, no studies have examined the interplay between the OLCN and local tissue composition or mechanics in a diabetic state, and the work presented here is a novel contribution (Figure 6, Supplementary Table 3). The strongest relationships between the OLCN parameters and local measures were for mechanical properties measured via nanoindentation. The number of branches near the lacunae explained 22% of the intracortical elastic work and 30% of the perilacunar elastic work (Figure 6). Similarly, a decrease in elastic modulus occurs contemporaneous to an increase in canalicular area with lactation.^32^ From a structure–function perspective, an increase in elastic work with fewer number of branches or less void space is logical, as an increased number of canaliculi (a more open system or increased void fraction) would concentrate local stresses and increase plastic deformation. An increased number of canaliculi could also potentially provide better stress dissipation and distribution, and a higher count of canaliculi might indicate a more actively remodeled “newer” bone matrix. In a TGF-β knockout model, a reduction in canalicular length was observed and coincided with a lower work of fracture, bending modulus, yield stress, supporting the notion that reduced OLCN architecture (ie, a reduction in canalicular branch number and length) contributes to worse mechanical outcomes.^33^ More broadly, the association between network parameters and local mechanics suggests that nanoindentation may be reliant on the underlying OLCN, and the OLCN should be considered when interpreting nanoindentation results, not just material-level properties, or tissue anisotropy.

It was hypothesized that compromises to the OLCN would decrease bone formation and affect local bone composition and mechanical properties. Significant correlations between OLCN measures and tissue composition were also detected. The number of branches near the lacunae explained 12% of the variation in intracortical proline/hydroxyproline and 16% in perilacunar proline/hydroxyproline. Overall for both the local compositional and mechanical properties—the measures taken at the perilacunar site (< 5 μm from the lacunae wall) were better explained by OLCN measures than intracortical. This makes sense when considering that perilacunar measures would be most dependent on osteocyte behavior, and the perturbations to the OLCN would be indicative of altered network maintenance under diabetic conditions. For local measurements, mean node degree was negatively associated with Raman MMR at the intracortical and perilacunar sites (data not shown). However, at the whole-bone level, a denser network was associated with an increased cortical BMD (Supplementary Table 2). This is nonintuitive, as a denser network would indicate greater OLCN void space and less mineral; however, this does align with previous associations between a more dense network and higher calcium.^34^ At the local level, the 1660/1690 cm^−1^ ratio negatively correlated with average branch tortuosity for the perilacunar region only (Supplementary Table 3). Spectroscopic measurements of the 1660/1690 cm^−1^ ratio have been correlated with direct measures of the ratio of mature trivalent collagen cross-link pyridinoline to immature divalent collagen cross-links via HPLC.^25^^,^^26^^,^^35^^,^^36^ The interpretation of Amide I subbands in Raman may not directly follow from FTIR.^29^ The Raman 1660/1690 cm^−1^ ratio may also be influenced by other features detected in the Amide I band. In Raman spectroscopy, the Amide I 1660 cm^−1^ subband is also indicative of disordered random coils,^37^ whereas the 1690 cm^−1^ subband is also linked to disordered secondary structures, including beta-turns^28^^,^^37^ or the absence of hydrogen bonds.^29^^,^^38^

Although osteocytes can remodel their local environment, perturbations to the composition and mechanical properties of the local surrounding matrix with diabetes may affect the fluid flow and shear stresses experienced by the osteocyte.^39^^,^^40^ Whether these changes to the perilacunar material composition are a cause or byproduct of an osteocyte’s response to diabetes is unclear. Computational studies have demonstrated how dependent the local stresses experienced by osteocytes are on the OLCN, creating models of fluid flow on individual osteocyte-canaliculi units, multicell, or organ-level units.^22^^,^^41–44^ Reduced lacunae and canalicular volume lower the osteocyte-experienced shear stress, while changes to tortuosity have a minimal impact on shear stress.^22^ It is reasonable then to assume that the reduced peripheral branches and decreased void fraction observed after 7 wk of STZ-induced hyperglycemia would reduce the shear stresses experienced by the osteocyte. Diabetic animal models have demonstrated a decreased anabolic response to loading in vivo.^45^^,^^46^ A sparser OLCN network, causing a reduction in shear stresses experienced by osteocytes, is a possible mechanism of action for the in vivo reduction in the anabolic response to loading.

The animals in this study were near skeletal maturity, as there was a reduction in the BFR (Figure 3) relative to BL mice. The lack of new tissue suggests that few osteocytes were actively embedding under the 3- or 7-wk diabetic conditions, and those that would be newly embedded nearest to the endosteal or periosteal surface were excluded from the analyses. These conditions narrow the study to the effect of diabetes on the maintenance and remodeling of the OLCN by already embedded osteocytes. In vitro studies have shown decreased expression of Connexin 43 in MLO-Y4 cells and primary osteocytes from animals with T2D.^47^ Further glucose and AGEs can induce osteocyte apoptosis and increase sclerostin expression.^13^^,^^48^ Activation of the receptor for AGEs (RAGE) increases apoptosis in MLO-Y4 cells,^49^ as well as modulating expressions of sclerostin and RANKL, both critical for osteoblasts and osteoclasts.^50^ However, in the same animals used in this study, no difference in RAGE expression was found in intracortical osteocytes.^6^ In vivo models of diabetes have also demonstrated an accumulation of senescent osteocytes with a proinflammatory signature.^51^ The peripheral OLCN being more affected suggests that osteocytes are unable to maintain the distant canaliculi and may be withdrawing. The mechanism for this is unclear, but as connexin 43 expression is diminished in MLO-Y4 cells and primary osteocytes from animals with T2D,^47^ there may be less availability of proteins and signaling factors necessary for branch maintenance, and the peripheral would be preferentially affected.

When comparing results from different animal studies, especially those examining age-dependent variables such as OLCN measures and tissue composition, the timing of disease onset is critical. Substantive bone formation only occurred in baseline mice (Figure 3). OLCN parameters explained ~20% of the variance in bone formation (Figure 4), but the OLCN may be more predictive in animals with a wider range of bone formation or resorption, such as mice undergoing rapid growth or subjected to exercise or other loading conditions. OLCN parameters did explain high levels of variance in bone morphology and mechanical properties, with the number of nodes explaining 45% of the variance in cortical bone volume fraction, 35% of cortical BMD, and 31% of ultimate load (Figure 5). D7 mice demonstrated significantly lower cortical BV/TV, BMD, and cortical thickness relative to BL, with significant diabetic factor effects for BV/TV, BMD, and cortical thickness.^6^ The predictive power of the OLCN over whole-bone measures like BV/TV suggests the OLCN influences perturbations to whole-bone geometry or OLCN changes occur contemporaneously to whole-bone changes. The OLCN was more predictive of whole-bone cortical morphology and ultimate load compared to bone formation, suggesting that the OLCN architecture is more strongly related to the remodeling or regulation of cross-sectional bone geometry.

No differences were detected in lacunae density, sphericity, or volume fraction with diabetes (Supplementary Table 1), suggesting that the peripheral changes in the OLCN network and topology precede alterations to lacunae density and morphology. The lack of difference in lacunae morphology and density was surprising, as studies with T1D Akita mice demonstrate a decreased lacunar density^14^ and mice fed a high-fat diet for 24 wk exhibit increased osteocyte volume.^5^ It is possible that differences in lacunae morphology and density occur after the deleterious effect of diabetes on the periphery of the OLCN and that the dendrites or canalicular morphology were more sensitive to the disease. This scenario is similar to the time course of reduced dendrite connectivity prior to a reduction in osteocyte number with age.^52^

This study focused on the cortical compartment of murine bone. Murine bone does not contain Haversian systems, whereas human bone does, with the osteocytes and lacunae arranged in a ring-like pattern around the Haversian canals.^53^ In murine bone, osteocytes are arranged around the marrow cavity similarly, but less concentrically than around the Haversian canals in human bone. The ranges of canalicular density and d_LC_ reported here (Figure 2D–F) are similar to previously characterized murine bone.^19^ Yet these murine canalicular densities are lower than that of published studies on human cadaveric bone that underwent a similar rhodamine staining protocol.^54^ Few studies have examined the parameters of the OLCN in human tissue, and caution should be taken when comparing the network parameters to parameters measured with different techniques (silver nitrate staining), across different skeletal sites, or even regions within the same cross-section. Although the results herein are suggestive, future studies should verify that diabetes-related changes to the osteocyte network exist in human tissue.

A limitation of this study is a reliance on the OLCN rather than examining the osteocytes themselves. Many studies have examined the effect of diabetes and/or hyperglycemia on MLO-Y4 cells and primary osteocytes. This work was focused on exploring the effect of diabetes on the OLCN and its relationship with local composition and mechanics, addressing a clear knowledge gap. Osteocytes are critical for maintaining their perilacunar environment, and their own network to properly function by remodeling and responding to mechanical stimuli. Understanding this critical component of skeletal health is necessary for elucidating the mechanisms of diabetic skeletal fragility.

The focus of this study was on the effect of hyperglycemia on the OLCN. To accomplish this, the consecutive low-dose STZ protocol was followed.^55^ This is not a direct model of the autoimmune destruction of pancreatic beta-cells that occurs with T1D. Hyperglycemia was confirmed via fasting blood glucose and endpoint HbA1c. Further characterization of the disease state could be accomplished by glucose or insulin tolerance testing. Future studies that examine the effect of loading or exercise on diabetic skeletal fragility and the OLCN should use these additional insulin/glucose tolerance tests to assess the disease state, as exercise increases glucose tolerance^56^ and insulin sensitivity^57^ in STZ-treated animals. Additionally, this study was limited to the use of male mice. Female mice are more resistant to streptozotocin-induced hyperglycemia,^15^^,^^16^ and their use would lessen disease severity or require the addition of a high-fat diet,^58^ increased STZ dose,^55^ or another diabetic animal model. Future investigation of sex-based differences in the OLCN, perilacunar composition, and mechanics is warranted.

The results presented herein support that the OLCN is disrupted with diabetes—through a reduction in node number and peripheral branching. Most novel, this study also demonstrated that OLCN measures offer predictive and explanatory power in the reduced bone formation, whole-bone geometry and mechanics, local mechanics, and perturbed local composition that occurs with diabetes. However, other potential explanations or confounding variables may exist. Measures of osteocyte activity that affect both OLCN architecture and maintenance of the local surrounding matrix may drive some of the relationships presented herein. Other potential mechanisms that should be explored include the role of microdamage accumulation, interstitial water content, and tissue anisotropy.

## Conclusions

This study demonstrates that the OLCN is reduced in STZ-induced diabetes. Specifically, in diabetes, there were fewer third-order through fifth-order branches surrounding individual lacunae, and branch tortuosity, total number of nodes, void fraction, and canalicular density all decreased. These compromises to the osteocyte network occurred without any changes in lacunae density or morphology. Most of these differences also occurred prior to a reduction in bone formation rate. Taken together, the data suggest a temporal sequence of perilacunar remodeling with diabetes that initiates with OLCN alterations prior to any reduction in bone formation by histomorphometry. The total number of nodes in the network positively correlated with bone formation, cortical bone fraction, cortical bone mineral density, and ultimate load, highlighting the importance of the OLCN in driving whole-bone properties. At the local level, the number of branches surrounding individual lacunae negatively correlated with elastic work and weakly correlated to proline/hydroxyproline at the intracortical and perilacunar regions, highlighting the complex interplay between the OLCN and the local composition and mechanics in the context of diabetes.

## Supplementary Material

Supplemental_Material_10-20-2023_ziad017

## Data Availability

The data underlying this article will be shared on reasonable request to the corresponding author.
