# Supplementary material for: Peripheral canalicular branching is decreased in streptozotocin-induced diabetes and correlates with decreased whole-bone ultimate load and perilacunar elastic work
Source: JBMR Plus. 2024 Jan 4;8(3):ziad017. doi: 10.1093/jbmrpl/ziad017 (PMC10945723; doi:10.1093/jbmrpl/ziad017)
Supplement: Supplemental_Material_10-20-2023_ziad017 [file supplemental_material_10-20-2023_ziad017.docx]

**
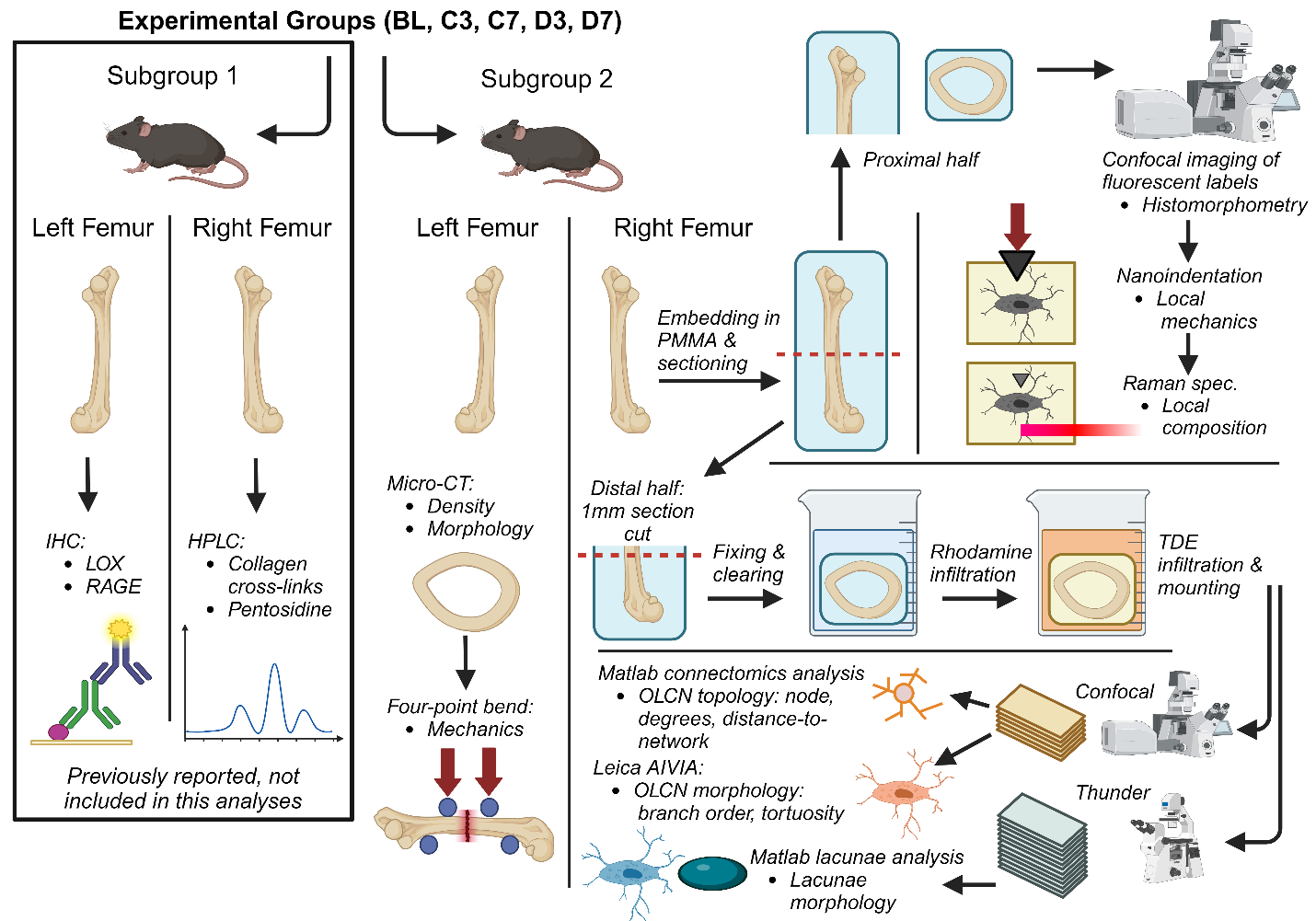
**

**Supplemental Figure 1:** Tissue distribution & analysis workflow of baseline (BL), control (C3, C7) and diabetic (D3, D7) groups. Only tissues from subgroup 2 are reported in this manuscript, those from subgroup 1 were previously published (Bolger *et al.*, *Calcified Tissue International*, 2023).

**Supplemental Table 1: Lacunae Morphology** Data are presented as mean (standard deviation) for each group for baseline (BL), 3-week control (C3), 3-week diabetic (D3), 7-week control (C7) and 7-week diabetic (D7). Two-way ANOVA carried out for C3, D3, C7, and D7 and significance (*p* < 0.05) between groups after Tukey’s post-hoc revealed no differences between groups. No significant factor effects (*p* < 0.05) were detected for diabetic status (D), time (T) and diabetic status-time interaction (DxT). One-way ANOVA with least-squares difference post-hoc was carried out to compare for significant differences relative to BL indicated by **&** (*p* < 0.05).
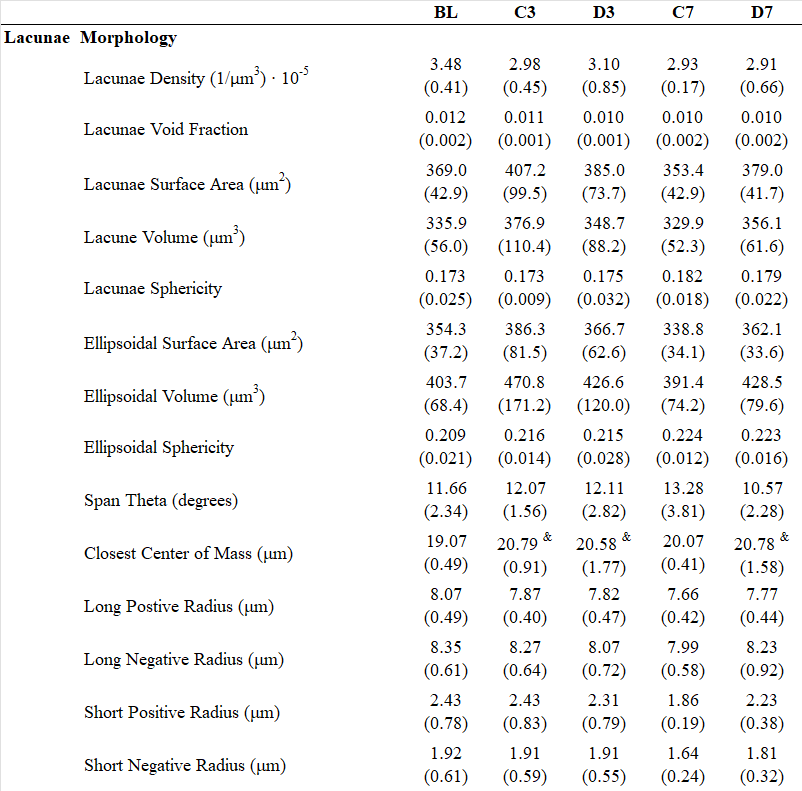


**Supplemental Table 2:** Pearson correlation coefficients between osteocyte lacuno-canalicular parameters and whole-bone measures of cortical morphology and mechanical properties. Only significant correlations (*p* < 0.05) are included in the table.

**Supplemental Table 3:** Pearson correlation coefficients between osteocyte lacuno-canalicular parameters and local Raman spectroscopic and nanoindentation measures of interest. Intracortical measurements were taken > 20 μm from any visible lacunae. Perilacunar measurements were taken < 5 μm from the lacunae wall. Only significant correlations (*p* < 0.05) are included in the table..
